# 5-Benzoyl-2-(1*H*-indol-3-yl)-4-[4-(propan-2-yl)phen­yl]-4,5-dihydro­furan-3-carbonitrile

**DOI:** 10.1107/S1600536812046764

**Published:** 2012-11-24

**Authors:** V. Rajni Swamy, R. V. Krishnakumar, N. Srinivasan, P. Gunasekaran, S. Perumal

**Affiliations:** a Department of Physics, Thiagarajar College, Madurai 625 009, India; b School of Chemistry, Madurai Kamaraj University, Madurai 625 021, India

## Abstract

In the title compound, C_29_H_24_N_2_O_2_, the hydrofuran ring is twisted with puckering parameters *Q* = 0.1553 (16) Å and ϕ = 305.0 (6)°. In the crystal, the graph-set motifs of the inter­action pattern are an *R*_2_^2^(16) motif involving dimers through N—H⋯N hydrogen bonds across centres of inversion and a *C*(6) motif through C—H⋯O hydrogen-bond between glide-related mol­ecules. Together, these generate [101] ladder-like chains.

## Related literature

For related structures, see: Suresh *et al.* (2012*a* ▶,*b* ▶,*c* ▶). For discussion on aromatic inter­actions, see: Bloom & Wheeler (2011 ▶); Martinez & Iverson (2012 ▶). For graph-set motifs, see: Bernstein *et al.* (1995 ▶). For puckering analysis, see: Cremer & Pople (1975 ▶).
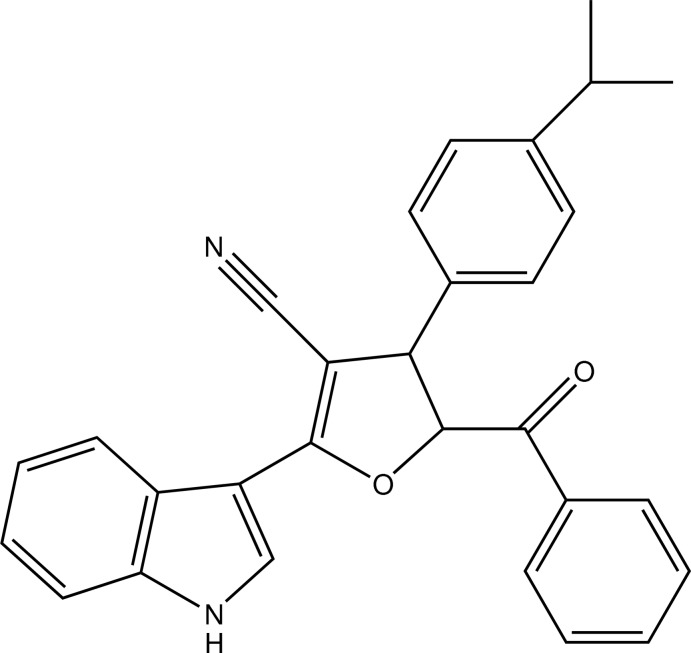


## Experimental

### 

#### Crystal data

C_29_H_24_N_2_O_2_
*M* *_r_* = 432.50
Monoclinic, 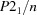

*a* = 17.1073 (4) Å
*b* = 8.1230 (2) Å
*c* = 17.6160 (4) Å
β = 110.325 (1)°
*V* = 2295.55 (9) Å^3^
*Z* = 4
Mo *K*α radiation
μ = 0.08 mm^−1^
*T* = 298 K
0.30 × 0.24 × 0.18 mm

#### Data collection

Bruker SMART APEXII CCD diffractometer
Absorption correction: multi-scan (*SADABS*; Bruker, 2009 ▶) *T*_min_ = 0.978, *T*_max_ = 0.986
21711 measured reflections
4455 independent reflections
2979 reflections with *I* > 2σ(*I*)
*R*_int_ = 0.031

#### Refinement

*R*[*F* ^2^ > 2σ(*F* ^2^)] = 0.040
*wR*(*F* ^2^) = 0.115
*S* = 1.03
4455 reflections
313 parameters
H atoms treated by a mixture of independent and constrained refinement
Δρ_max_ = 0.14 e Å^−3^
Δρ_min_ = −0.15 e Å^−3^

### 

Data collection: *APEX2* (Bruker, 2009 ▶); cell refinement: *SAINT* (Bruker, 2009 ▶); data reduction: *SAINT*; program(s) used to solve structure: *SHELXS97* (Sheldrick, 2008 ▶); program(s) used to refine structure: *SHELXL97* (Sheldrick, 2008 ▶); molecular graphics: *PLATON* (Spek, 2009 ▶); software used to prepare material for publication: *SHELXL97*.

## Supplementary Material

Crystal structure: contains datablock(s) I, global. DOI: 10.1107/S1600536812046764/rz5022sup1.cif

Structure factors: contains datablock(s) I. DOI: 10.1107/S1600536812046764/rz5022Isup2.hkl

Supplementary material file. DOI: 10.1107/S1600536812046764/rz5022Isup3.cml

Additional supplementary materials:  crystallographic information; 3D view; checkCIF report

## Figures and Tables

**Table 1:** Hydrogen-bond geometry (Å, °)

*D*—H⋯*A*	*D*—H	H⋯*A*	*D*⋯*A*	*D*—H⋯*A*
N2—H2⋯N1^i^	0.91 (2)	2.09 (2)	2.973 (2)	165.2 (17)
C56—H56⋯O2^ii^	0.978 (15)	2.411 (16)	3.371 (2)	166.9 (13)

Symmetry codes: (i) 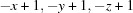; (ii) 
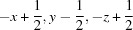
.

**Fig. 1.**
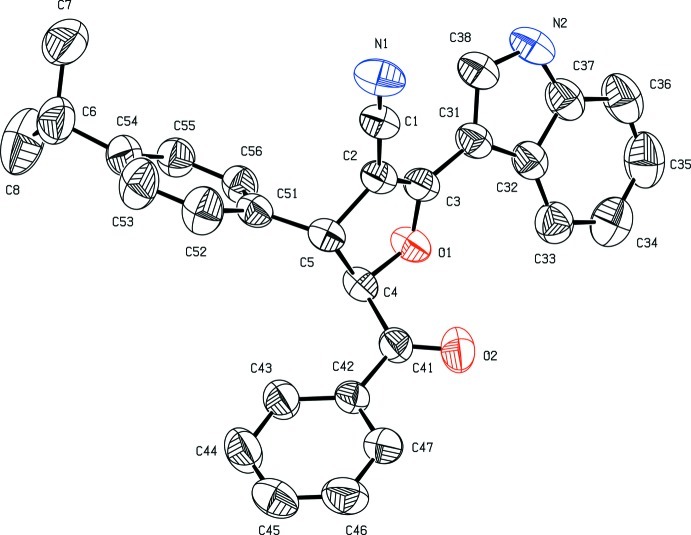
The molecular structure of the title compound showing displacement ellipsoids drawn at the 50% probability level. Hydrogen atoms have been omitted for clarity.

**Fig. 2.**
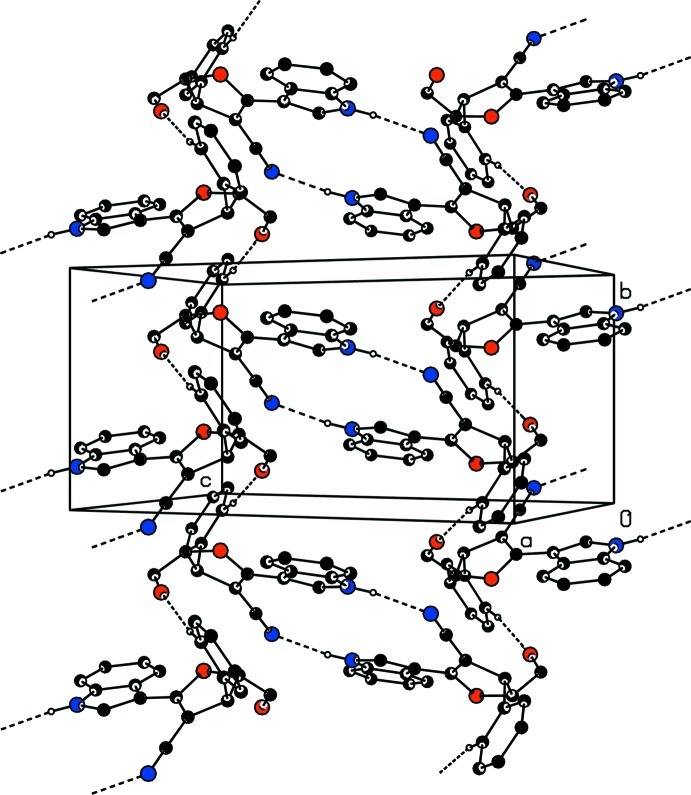
The R22(16) N—H···N mediated centrosymmetric dimeric units linked via C(6) C—H···O linkages to form a supramolecular ladder. Non-participating groups and H-atoms are omitted for clarity.

**Fig. 3.**
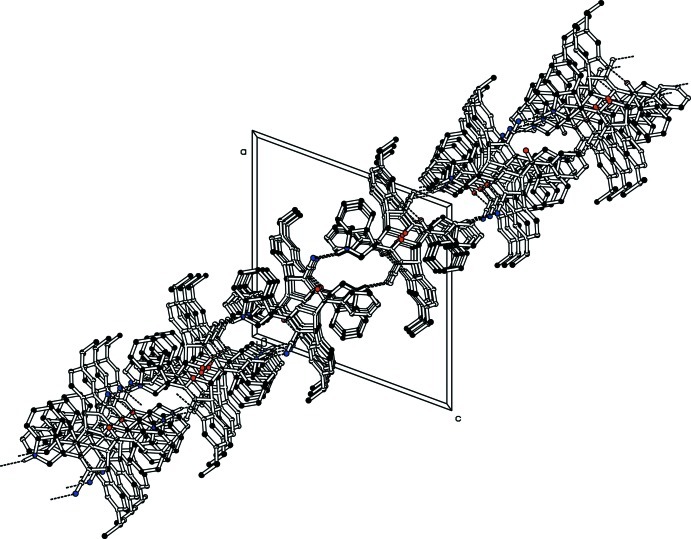
Perspective view of supramolecular ladders parallel to the b axis extending along the [101] direction. Non-participating groups and H-atoms are omitted for clarity.

## supplementary crystallographic information

### Comment

In the molecule of the title compound (Fig. 1), the puckering (Cremer & Pople, 1975) of the furan ring is twisted (^4^T_5_) about the C4—C5 bond with Q = 0.1553 (16) Å and φ = 305.0 (6)°. The geometry of the N—H···N and C—H···O interactions that characterize the crystal packing (Table 1) are similar to its 4-phenyl analogue (Suresh *et al.*, 2012*a*). The graph-set motifs (Bernstein *et al.*, 1995) that characterize the intermolecular interaction pattern are a *R*^2^_2_(16) involving dimers through N—H···N hydrogen bonds across centres of inversion and a *C*(6) motif through C—H···O hydrogen-bond between glide-related molecules (Fig. 2). The crystal structure may be visualized as zero-dimensional N—H···N mediated centrosymmetric dimeric units linked *via* C—H···O linkages to form a supramolecular ladder parallel to the *b* axis that extends along the [101] direction (Fig. 3). The non-covalent crystal packing interactions are closely related to that observed in the 4-phenyl analogue and distinctly differs from those of the 4-methylphenyl (Suresh *et al.*, 2012*b*) and 4-bromophenyl (Suresh *et al.*, 2012*c*) analogues. The intramolecular C—H···O hydrogen bond generates a *S*(6) motif and its geometry is similar to those observed in all of the above analogues. No significant weak non-covalent crystal packing interactions involving centroids of planar rings are observed. Though it has become an acceptable norm in describing weak non-covalent interactions in terms of C—H···π and π···π interactions, some recent views (Martinez & Iverson, 2012; Bloom & Wheeler, 2011) on these interactions calls for a careful analysis while describing them in crystal structures.

The crystal structure of the title compound together with those of its analogues demonstrate the effect of substituents in drastically altering the interaction patterns while retaining the crystal system and lattice type.

### Experimental

To a stirred mixture of 2-(1*H*-indole-3-carbonyl)-3-(4-isopropylphenyl)acrylonitrile (1.0 eq) and phenacylpyridinium bromide (1.0 eq) in water (10 ml) was added drop wise triethylamine (0.25 eq) at room temperature. The resulting clear solution, that slowly became turbid, was stirred at room temperature for 1.5 h. Then the separated free flowing solid was filtered and washed with methanol (3 ml) to afford the compound as pale yellow solid. Yield 87%; m.p. 508 K.

### Refinement

All hydrogen atoms except those participating in the hydrogen-bonding were included into the model at geometrically calculated positions (C—H target distance 0.96 Å for methyl hydrogen atoms, 0.93 Å for all others) and refined using a riding model. The torsion angle of the methyl groups involving C7 and C8 were allowed to refine. The *U_iso_* values of all hydrogen atoms were constrained to 1.2 times *U_eq_* (1.5 times for methyl H atoms) of the respective atom to which the hydrogen atom binds. The positions of the hydrogen atoms bound to N2, C33 and C56 were allowed to refine isotropically. A reflection (1 0 1) partially obstructed by the primary beam stop was omitted from the refinement.

### Crystal data

**Table:**

C_29_H_24_N_2_O_2_	*F*(000) = 912
*M**_r_* = 432.50	*D*_x_ = 1.251 Mg m^−^^3^
Monoclinic, *P*2_1_/*n*	Mo *K*α radiation, λ = 0.71073 Å
Hall symbol: -P 2yn	Cell parameters from 4455 reflections
*a* = 17.1073 (4) Å	θ = 2.5–25.9°
*b* = 8.1230 (2) Å	µ = 0.08 mm^−^^1^
*c* = 17.6160 (4) Å	*T* = 298 K
β = 110.325 (1)°	Block, yellow
*V* = 2295.55 (9) Å^3^	0.30 × 0.24 × 0.18 mm
*Z* = 4	

### Data collection

**Table:**

Bruker SMART APEXII CCD diffractometer	4455 independent reflections
Radiation source: fine-focus sealed tube	2979 reflections with *I* > 2σ(*I*)
Graphite monochromator	*R*_int_ = 0.031
ω and φ scans	θ_max_ = 25.9°, θ_min_ = 2.5°
Absorption correction: multi-scan (*SADABS*; Bruker, 2009)	*h* = −21→20
*T*_min_ = 0.978, *T*_max_ = 0.986	*k* = −9→9
21711 measured reflections	*l* = −21→21

### Refinement

**Table:**

Refinement on *F*^2^	Secondary atom site location: difference Fourier map
Least-squares matrix: full	Hydrogen site location: inferred from neighbouring sites
*R*[*F*^2^ > 2σ(*F*^2^)] = 0.040	H atoms treated by a mixture of independent and constrained refinement
*wR*(*F*^2^) = 0.115	*w* = 1/[σ^2^(*F*_o_^2^) + (0.0478*P*)^2^ + 0.3844*P*] where *P* = (*F*_o_^2^ + 2*F*_c_^2^)/3
*S* = 1.03	(Δ/σ)_max_ < 0.001
4455 reflections	Δρ_max_ = 0.14 e Å^−^^3^
313 parameters	Δρ_min_ = −0.15 e Å^−^^3^
0 restraints	Extinction correction: *SHELXL97* (Sheldrick, 2008), Fc^*^=kFc[1+0.001xFc^2^λ^3^/sin(2θ)]^-1/4^
Primary atom site location: structure-invariant direct methods	Extinction coefficient: 0.0037 (8)

### Special details

**Table:**

Geometry. All e.s.d.'s (except the e.s.d. in the dihedral angle between two l.s. planes) are estimated using the full covariance matrix. The cell e.s.d.'s are taken into account individually in the estimation of e.s.d.'s in distances, angles and torsion angles; correlations between e.s.d.'s in cell parameters are only used when they are defined by crystal symmetry. An approximate (isotropic) treatment of cell e.s.d.'s is used for estimating e.s.d.'s involving l.s. planes.
Refinement. Refinement of *F*^2^ against ALL reflections. The weighted *R*-factor *wR* and goodness of fit *S* are based on *F*^2^, conventional *R*-factors *R* are based on *F*, with *F* set to zero for negative *F*^2^. The threshold expression of *F*^2^ > σ(*F*^2^) is used only for calculating *R*-factors(gt) *etc*. and is not relevant to the choice of reflections for refinement. *R*-factors based on *F*^2^ are statistically about twice as large as those based on *F*, and *R*- factors based on ALL data will be even larger.

### Fractional atomic coordinates and isotropic or equivalent isotropic displacement parameters (Å^2^)

**Table:**

	*x*	*y*	*z*	*U*_iso_*/*U*_eq_	
O1	0.27185 (7)	0.17860 (13)	0.26234 (6)	0.0553 (3)	
O2	0.14529 (8)	0.34499 (18)	0.16547 (7)	0.0792 (4)	
N1	0.49191 (10)	0.5602 (2)	0.31131 (9)	0.0752 (5)	
N2	0.40406 (11)	0.3263 (2)	0.52408 (9)	0.0694 (5)	
H2	0.4434 (13)	0.352 (3)	0.5722 (13)	0.088 (6)*	
C38	0.41572 (11)	0.3360 (2)	0.45193 (10)	0.0611 (5)	
H38	0.4652	0.3669	0.4451	0.073*	
C37	0.32392 (12)	0.2788 (2)	0.51144 (10)	0.0623 (5)	
C36	0.28453 (16)	0.2536 (3)	0.56731 (12)	0.0808 (6)	
H36	0.3124	0.2690	0.6224	0.097*	
C35	0.20349 (17)	0.2056 (3)	0.53802 (15)	0.0907 (7)	
H35	0.1756	0.1862	0.5740	0.109*	
C34	0.16088 (14)	0.1847 (3)	0.45517 (14)	0.0828 (6)	
H34	0.1051	0.1537	0.4371	0.099*	
C33	0.20012 (12)	0.2091 (2)	0.39978 (12)	0.0640 (5)	
H33	0.1721 (10)	0.194 (2)	0.3426 (10)	0.063 (5)*	
C32	0.28362 (11)	0.25594 (19)	0.42818 (9)	0.0527 (4)	
C31	0.34395 (10)	0.29363 (19)	0.39077 (9)	0.0510 (4)	
C3	0.33352 (9)	0.28117 (19)	0.30620 (9)	0.0481 (4)	
C2	0.37493 (10)	0.34966 (19)	0.26182 (9)	0.0486 (4)	
C1	0.43942 (11)	0.4654 (2)	0.28977 (9)	0.0544 (4)	
C5	0.34552 (9)	0.27757 (19)	0.17735 (9)	0.0477 (4)	
H5	0.3302	0.3663	0.1371	0.057*	
C4	0.26595 (9)	0.18830 (19)	0.17900 (8)	0.0483 (4)	
H4	0.2641	0.0769	0.1571	0.058*	
C41	0.18682 (10)	0.2796 (2)	0.13117 (9)	0.0505 (4)	
C42	0.16376 (9)	0.28783 (19)	0.04210 (9)	0.0472 (4)	
C43	0.20139 (11)	0.1912 (2)	0.00015 (10)	0.0613 (5)	
H43	0.2440	0.1194	0.0281	0.074*	
C44	0.17592 (13)	0.2007 (3)	−0.08313 (11)	0.0751 (6)	
H44	0.2012	0.1348	−0.1111	0.090*	
C45	0.11360 (13)	0.3069 (2)	−0.12478 (11)	0.0725 (5)	
H45	0.0966	0.3129	−0.1809	0.087*	
C46	0.07638 (12)	0.4040 (3)	−0.08392 (11)	0.0741 (5)	
H46	0.0343	0.4767	−0.1121	0.089*	
C47	0.10122 (11)	0.3943 (2)	−0.00096 (10)	0.0633 (5)	
H47	0.0755	0.4604	0.0265	0.076*	
C51	0.41055 (9)	0.16822 (19)	0.16311 (9)	0.0486 (4)	
C52	0.44866 (12)	0.2118 (2)	0.10860 (11)	0.0718 (5)	
H52	0.4313	0.3059	0.0772	0.086*	
C53	0.51201 (13)	0.1176 (3)	0.10024 (12)	0.0803 (6)	
H53	0.5365	0.1498	0.0630	0.096*	
C54	0.54025 (11)	−0.0221 (2)	0.14503 (10)	0.0631 (5)	
C55	0.49997 (10)	−0.0680 (2)	0.19778 (10)	0.0579 (4)	
H55	0.5163	−0.1640	0.2280	0.070*	
C56	0.43633 (10)	0.0251 (2)	0.20650 (9)	0.0518 (4)	
H56	0.4086 (9)	−0.0097 (19)	0.2440 (9)	0.059 (4)*	
C6	0.61471 (13)	−0.1150 (3)	0.14021 (12)	0.0827 (6)	
H6	0.6245	−0.0765	0.0916	0.099*	
C7	0.69188 (13)	−0.0692 (4)	0.21299 (13)	0.1129 (9)	
H7A	0.6812	−0.0901	0.2621	0.169*	
H7B	0.7043	0.0453	0.2101	0.169*	
H7C	0.7385	−0.1343	0.2123	0.169*	
C8	0.60164 (18)	−0.2977 (3)	0.1318 (2)	0.1309 (11)	
H8A	0.5946	−0.3408	0.1797	0.196*	
H8B	0.6492	−0.3484	0.1244	0.196*	
H8C	0.5528	−0.3208	0.0857	0.196*	

### Atomic displacement parameters (Å^2^)

**Table:**

	*U*^11^	*U*^22^	*U*^33^	*U*^12^	*U*^13^	*U*^23^
O1	0.0631 (7)	0.0602 (7)	0.0400 (6)	−0.0138 (6)	0.0145 (5)	−0.0035 (5)
O2	0.0800 (9)	0.1019 (10)	0.0621 (8)	0.0212 (8)	0.0331 (7)	−0.0097 (7)
N1	0.0769 (11)	0.0719 (11)	0.0629 (10)	−0.0211 (9)	0.0067 (8)	−0.0012 (8)
N2	0.0827 (12)	0.0747 (11)	0.0406 (9)	−0.0067 (9)	0.0087 (8)	−0.0032 (7)
C38	0.0699 (11)	0.0623 (11)	0.0458 (10)	−0.0031 (9)	0.0134 (8)	−0.0008 (8)
C37	0.0830 (14)	0.0546 (10)	0.0484 (10)	0.0046 (10)	0.0217 (9)	0.0016 (8)
C36	0.1129 (18)	0.0812 (14)	0.0558 (12)	0.0064 (13)	0.0389 (12)	0.0030 (10)
C35	0.1152 (19)	0.0943 (17)	0.0842 (16)	0.0108 (15)	0.0619 (15)	0.0097 (13)
C34	0.0788 (14)	0.0842 (15)	0.0955 (17)	0.0055 (12)	0.0432 (13)	0.0062 (12)
C33	0.0687 (12)	0.0613 (11)	0.0629 (12)	0.0076 (9)	0.0242 (10)	0.0011 (9)
C32	0.0651 (11)	0.0443 (9)	0.0472 (9)	0.0069 (8)	0.0176 (8)	0.0011 (7)
C31	0.0603 (10)	0.0470 (9)	0.0413 (9)	0.0028 (8)	0.0120 (7)	0.0004 (7)
C3	0.0512 (9)	0.0442 (9)	0.0435 (9)	0.0018 (7)	0.0094 (7)	−0.0024 (7)
C2	0.0511 (9)	0.0465 (9)	0.0438 (8)	−0.0020 (8)	0.0107 (7)	−0.0027 (7)
C1	0.0584 (10)	0.0531 (10)	0.0450 (9)	−0.0036 (9)	0.0095 (8)	−0.0004 (8)
C5	0.0520 (9)	0.0473 (9)	0.0402 (8)	−0.0047 (7)	0.0115 (7)	0.0002 (7)
C4	0.0556 (9)	0.0490 (9)	0.0394 (8)	−0.0055 (7)	0.0155 (7)	−0.0059 (7)
C41	0.0511 (9)	0.0531 (9)	0.0485 (9)	−0.0050 (8)	0.0188 (8)	−0.0101 (7)
C42	0.0453 (9)	0.0486 (9)	0.0460 (9)	−0.0057 (7)	0.0137 (7)	−0.0074 (7)
C43	0.0677 (11)	0.0652 (11)	0.0506 (10)	0.0086 (9)	0.0199 (8)	−0.0026 (8)
C44	0.0912 (14)	0.0846 (14)	0.0548 (11)	0.0057 (12)	0.0320 (11)	−0.0082 (10)
C45	0.0856 (14)	0.0795 (14)	0.0462 (10)	−0.0104 (11)	0.0149 (10)	0.0021 (10)
C46	0.0728 (12)	0.0755 (13)	0.0581 (12)	0.0063 (10)	0.0030 (10)	0.0016 (10)
C47	0.0568 (10)	0.0696 (12)	0.0568 (11)	0.0039 (9)	0.0110 (8)	−0.0088 (9)
C51	0.0525 (9)	0.0532 (9)	0.0392 (8)	−0.0068 (8)	0.0148 (7)	−0.0013 (7)
C52	0.0848 (13)	0.0751 (13)	0.0671 (12)	0.0079 (11)	0.0410 (11)	0.0220 (10)
C53	0.0857 (14)	0.1008 (16)	0.0742 (13)	0.0119 (12)	0.0528 (11)	0.0227 (12)
C54	0.0628 (11)	0.0780 (13)	0.0539 (10)	0.0029 (10)	0.0271 (9)	−0.0005 (9)
C55	0.0620 (11)	0.0598 (11)	0.0529 (10)	0.0034 (9)	0.0212 (8)	0.0046 (8)
C56	0.0579 (10)	0.0575 (10)	0.0445 (9)	−0.0049 (8)	0.0234 (8)	0.0026 (8)
C6	0.0760 (13)	0.1117 (18)	0.0713 (13)	0.0184 (13)	0.0393 (11)	0.0067 (12)
C7	0.0725 (15)	0.190 (3)	0.0792 (16)	0.0226 (16)	0.0300 (13)	0.0053 (16)
C8	0.125 (2)	0.110 (2)	0.182 (3)	0.0356 (18)	0.084 (2)	−0.005 (2)

### Geometric parameters (Å, º)

**Table:**

O1—C3	1.3557 (18)	C42—C43	1.381 (2)
O1—C4	1.4384 (17)	C43—C44	1.380 (2)
O2—C41	1.2043 (18)	C43—H43	0.9300
N1—C1	1.143 (2)	C44—C45	1.370 (3)
N2—C38	1.356 (2)	C44—H44	0.9300
N2—C37	1.366 (2)	C45—C46	1.365 (3)
N2—H2	0.91 (2)	C45—H45	0.9300
C38—C31	1.367 (2)	C46—C47	1.375 (2)
C38—H38	0.9300	C46—H46	0.9300
C37—C36	1.388 (3)	C47—H47	0.9300
C37—C32	1.399 (2)	C51—C56	1.377 (2)
C36—C35	1.358 (3)	C51—C52	1.381 (2)
C36—H36	0.9300	C52—C53	1.376 (3)
C35—C34	1.397 (3)	C52—H52	0.9300
C35—H35	0.9300	C53—C54	1.371 (3)
C34—C33	1.378 (3)	C53—H53	0.9300
C34—H34	0.9300	C54—C55	1.387 (2)
C33—C32	1.392 (2)	C54—C6	1.508 (3)
C33—H33	0.961 (16)	C55—C56	1.377 (2)
C32—C31	1.437 (2)	C55—H55	0.9300
C31—C3	1.441 (2)	C56—H56	0.978 (15)
C3—C2	1.344 (2)	C6—C8	1.501 (3)
C2—C1	1.402 (2)	C6—C7	1.533 (3)
C2—C5	1.513 (2)	C6—H6	0.9800
C5—C51	1.511 (2)	C7—H7A	0.9600
C5—C4	1.552 (2)	C7—H7B	0.9600
C5—H5	0.9800	C7—H7C	0.9600
C4—C41	1.516 (2)	C8—H8A	0.9600
C4—H4	0.9800	C8—H8B	0.9600
C41—C42	1.480 (2)	C8—H8C	0.9600
C42—C47	1.380 (2)		
			
C3—O1—C4	108.23 (11)	C43—C42—C41	122.63 (15)
C38—N2—C37	109.26 (16)	C44—C43—C42	120.21 (17)
C38—N2—H2	124.0 (13)	C44—C43—H43	119.9
C37—N2—H2	126.6 (12)	C42—C43—H43	119.9
N2—C38—C31	109.88 (16)	C45—C44—C43	120.31 (17)
N2—C38—H38	125.1	C45—C44—H44	119.8
C31—C38—H38	125.1	C43—C44—H44	119.8
N2—C37—C36	129.28 (18)	C46—C45—C44	120.01 (17)
N2—C37—C32	108.03 (15)	C46—C45—H45	120.0
C36—C37—C32	122.68 (19)	C44—C45—H45	120.0
C35—C36—C37	117.1 (2)	C45—C46—C47	119.89 (18)
C35—C36—H36	121.4	C45—C46—H46	120.1
C37—C36—H36	121.4	C47—C46—H46	120.1
C36—C35—C34	121.69 (19)	C46—C47—C42	120.99 (17)
C36—C35—H35	119.2	C46—C47—H47	119.5
C34—C35—H35	119.2	C42—C47—H47	119.5
C33—C34—C35	121.1 (2)	C56—C51—C52	117.70 (15)
C33—C34—H34	119.4	C56—C51—C5	121.09 (13)
C35—C34—H34	119.4	C52—C51—C5	121.15 (15)
C34—C33—C32	118.44 (19)	C53—C52—C51	120.76 (17)
C34—C33—H33	122.3 (10)	C53—C52—H52	119.6
C32—C33—H33	119.2 (10)	C51—C52—H52	119.6
C33—C32—C37	118.90 (16)	C54—C53—C52	122.15 (16)
C33—C32—C31	134.73 (15)	C54—C53—H53	118.9
C37—C32—C31	106.34 (15)	C52—C53—H53	118.9
C38—C31—C32	106.48 (14)	C53—C54—C55	116.79 (16)
C38—C31—C3	125.98 (15)	C53—C54—C6	121.17 (16)
C32—C31—C3	127.46 (15)	C55—C54—C6	121.95 (17)
C2—C3—O1	112.70 (13)	C56—C55—C54	121.53 (17)
C2—C3—C31	132.15 (15)	C56—C55—H55	119.2
O1—C3—C31	115.13 (13)	C54—C55—H55	119.2
C3—C2—C1	125.62 (14)	C51—C56—C55	121.00 (15)
C3—C2—C5	110.44 (13)	C51—C56—H56	118.8 (9)
C1—C2—C5	123.86 (14)	C55—C56—H56	120.2 (9)
N1—C1—C2	178.90 (18)	C8—C6—C54	113.64 (18)
C51—C5—C2	112.23 (12)	C8—C6—C7	112.2 (2)
C51—C5—C4	115.45 (13)	C54—C6—C7	109.29 (17)
C2—C5—C4	99.01 (11)	C8—C6—H6	107.1
C51—C5—H5	109.9	C54—C6—H6	107.1
C2—C5—H5	109.9	C7—C6—H6	107.1
C4—C5—H5	109.9	C6—C7—H7A	109.5
O1—C4—C41	109.06 (12)	C6—C7—H7B	109.5
O1—C4—C5	107.08 (11)	H7A—C7—H7B	109.5
C41—C4—C5	112.38 (12)	C6—C7—H7C	109.5
O1—C4—H4	109.4	H7A—C7—H7C	109.5
C41—C4—H4	109.4	H7B—C7—H7C	109.5
C5—C4—H4	109.4	C6—C8—H8A	109.5
O2—C41—C42	121.68 (15)	C6—C8—H8B	109.5
O2—C41—C4	120.35 (14)	H8A—C8—H8B	109.5
C42—C41—C4	117.96 (13)	C6—C8—H8C	109.5
C47—C42—C43	118.59 (15)	H8A—C8—H8C	109.5
C47—C42—C41	118.78 (14)	H8B—C8—H8C	109.5
			
C37—N2—C38—C31	−0.6 (2)	C2—C5—C4—O1	−15.46 (14)
C38—N2—C37—C36	−179.78 (19)	C51—C5—C4—C41	−135.75 (13)
C38—N2—C37—C32	0.6 (2)	C2—C5—C4—C41	104.28 (13)
N2—C37—C36—C35	−179.8 (2)	O1—C4—C41—O2	7.6 (2)
C32—C37—C36—C35	−0.3 (3)	C5—C4—C41—O2	−110.94 (17)
C37—C36—C35—C34	−1.0 (3)	O1—C4—C41—C42	−173.49 (12)
C36—C35—C34—C33	1.3 (3)	C5—C4—C41—C42	67.93 (17)
C35—C34—C33—C32	−0.2 (3)	O2—C41—C42—C47	10.5 (2)
C34—C33—C32—C37	−1.0 (3)	C4—C41—C42—C47	−168.32 (14)
C34—C33—C32—C31	−179.20 (18)	O2—C41—C42—C43	−168.36 (17)
N2—C37—C32—C33	−179.12 (16)	C4—C41—C42—C43	12.8 (2)
C36—C37—C32—C33	1.3 (3)	C47—C42—C43—C44	−0.6 (3)
N2—C37—C32—C31	−0.42 (19)	C41—C42—C43—C44	178.27 (16)
C36—C37—C32—C31	179.96 (16)	C42—C43—C44—C45	0.5 (3)
N2—C38—C31—C32	0.32 (19)	C43—C44—C45—C46	0.0 (3)
N2—C38—C31—C3	−176.61 (15)	C44—C45—C46—C47	−0.4 (3)
C33—C32—C31—C38	178.46 (19)	C45—C46—C47—C42	0.3 (3)
C37—C32—C31—C38	0.07 (18)	C43—C42—C47—C46	0.3 (3)
C33—C32—C31—C3	−4.7 (3)	C41—C42—C47—C46	−178.69 (16)
C37—C32—C31—C3	176.94 (15)	C2—C5—C51—C56	63.99 (19)
C4—O1—C3—C2	−5.21 (17)	C4—C5—C51—C56	−48.45 (19)
C4—O1—C3—C31	176.25 (13)	C2—C5—C51—C52	−113.25 (17)
C38—C31—C3—C2	−23.4 (3)	C4—C5—C51—C52	134.30 (16)
C32—C31—C3—C2	160.29 (17)	C56—C51—C52—C53	−2.1 (3)
C38—C31—C3—O1	154.78 (15)	C5—C51—C52—C53	175.27 (17)
C32—C31—C3—O1	−21.5 (2)	C51—C52—C53—C54	−0.1 (3)
O1—C3—C2—C1	177.33 (14)	C52—C53—C54—C55	2.1 (3)
C31—C3—C2—C1	−4.4 (3)	C52—C53—C54—C6	−174.53 (19)
O1—C3—C2—C5	−5.75 (18)	C53—C54—C55—C56	−1.9 (3)
C31—C3—C2—C5	172.48 (16)	C6—C54—C55—C56	174.67 (17)
C3—C2—C5—C51	−109.41 (15)	C52—C51—C56—C55	2.2 (2)
C1—C2—C5—C51	67.58 (19)	C5—C51—C56—C55	−175.12 (14)
C3—C2—C5—C4	12.92 (16)	C54—C55—C56—C51	−0.2 (3)
C1—C2—C5—C4	−170.09 (15)	C53—C54—C6—C8	−133.9 (2)
C3—O1—C4—C41	−108.29 (13)	C55—C54—C6—C8	49.6 (3)
C3—O1—C4—C5	13.55 (15)	C53—C54—C6—C7	99.9 (2)
C51—C5—C4—O1	104.52 (14)	C55—C54—C6—C7	−76.6 (2)

### Hydrogen-bond geometry (Å, º)

**Table:**

*D*—H···*A*	*D*—H	H···*A*	*D*···*A*	*D*—H···*A*
C33—H33···O1	0.961 (16)	2.569 (15)	3.081 (2)	113.5 (11)
N2—H2···N1^i^	0.91 (2)	2.09 (2)	2.973 (2)	165.2 (17)
C56—H56···O2^ii^	0.978 (15)	2.411 (16)	3.371 (2)	166.9 (13)

Symmetry codes: (i) −*x*+1, −*y*+1, −*z*+1; (ii) −*x*+1/2, *y*−1/2, −*z*+1/2.
